# Biomimetic Ketone Reduction by Disulfide Radical Anion

**DOI:** 10.3390/molecules26185429

**Published:** 2021-09-07

**Authors:** Sebastian Barata-Vallejo, Konrad Skotnicki, Carla Ferreri, Bronislaw Marciniak, Krzysztof Bobrowski, Chryssostomos Chatgilialoglu

**Affiliations:** 1 Istituto per la Sintesi Organica e la Fotoreattività (ISOF), Consiglio Nazionale delle Ricerche (CNR), Via P. Gobetti 101, 40129 Bologna, Italy; sebastian.barata@isof.cnr.it (S.B.-V.); carla.ferreri@isof.cnr.it (C.F.); 2 Departamento de Ciencias Químicas, Facultad de Farmacia y Bioquimíca, Universidad de Buenos Aires, Junin 954, Buenos Aires CP 1113, Argentina; 3 Institute of Nuclear Chemistry and Technology, Dorodna 16, 03-195 Warsaw, Poland; k.skotnicki@ichtj.waw.pl (K.S.); kris@ichtj.pl (K.B.); 4 Center for Advanced Technology, Adam Mickiewicz University, Uniwersytetu Poznanskiego 10, 61-614 Poznan, Poland; marcinia@amu.edu.pl

**Keywords:** biomimetic chemistry, cysteine, ketone reduction, free radicals, pulse radiolysis, kinetics

## Abstract

The conversion of ribonucleosides to 2′-deoxyribonucleosides is catalyzed by ribonucleoside reductase enzymes in nature. One of the key steps in this complex radical mechanism is the reduction of the 3′-ketodeoxynucleotide by a pair of cysteine residues, providing the electrons via a disulfide radical anion (RSSR^•−^) in the active site of the enzyme. In the present study, the bioinspired conversion of ketones to corresponding alcohols was achieved by the intermediacy of disulfide radical anion of cysteine (CysSSCys)^•−^ in water. High concentration of cysteine and pH 10.6 are necessary for high-yielding reactions. The photoinitiated radical chain reaction includes the one-electron reduction of carbonyl moiety by disulfide radical anion, protonation of the resulting ketyl radical anion by water, and H-atom abstraction from CysSH. The (CysSSCys)^•−^ transient species generated by ionizing radiation in aqueous solutions allowed the measurement of kinetic data with ketones by pulse radiolysis. By measuring the rate of the decay of (CysSSCys)^•−^ at λ_max_ = 420 nm at various concentrations of ketones, we found the rate constants of three cyclic ketones to be in the range of 10^4^–10^5^ M^−1^s^−1^ at ~22 °C.

## 1. Introduction

The comprehension of free radical reactivity taking place in naturally occurring processes can be very important for chemistry in two main ways: (1) inspiring new synthetic methods based on the same mechanisms that nature uses to prepare biomolecules, and (2) designing biomimetic models to simulate the free radical damages and to provide molecular libraries for mechanistic and biomarker discovery. Two successful examples of these approaches from our group are the syntheses of 5′,8-cyclopurine lesions [1,2,3] and mono-trans PUFA isomers [4,5,6]. Indeed, there are strict relationships between reactivity involving free radicals and processes taking place in nature [7]. Since biological reactivity occurs in an aqueous environment, free radical chemistry can become attractive for realizing eco-friendly and high-yielding methodologies.

**Figure 1:**
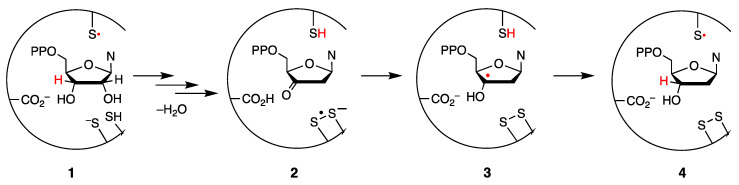
The transformation of ribonucleotides into 2′-ribonucleotides by RNR.

The radical chemistry associated with thiols (RSH) and disulfides (RSSR) plays important roles in nature, particularly in enzymes and proteins. One example is the conversion of ribonucleosides to 2′-deoxyribonucleosides, the monomers required for the construction of DNA, catalyzed by radical enzymes called ribonucleoside reductases (RNRs). There are three forms of the enzyme (classes I, II, and III) that are active in different species, with the former being active in eukaryotes and microorganisms, using ribonucleotide diphosphates as substrates [8,9,10]. An intriguing step in class I is the transfer from the tyrosyl radical to the thiol moiety in the active site where the ribonucleotide substrate waits for the conversion. Figure 1 summarizes the complex mechanism of this transformation, which initially involves a reversible 3′-hydrogen atom abstraction by the thiyl radical generated from the cysteine residues in the active site (**1**). There follows the elimination of water with translocation of the radical center to C2′ and quenching of the newly formed C2′ radical by another cysteine residue (**2**). This generates the disulfide radical anion (RSSR^•−^) that reduces the 3′-ketodeoxynucleotide (**2**→**3**) [11,12]. To complete the cycle, the C3′ radical abstracts the hydrogen from the initial cysteine residue (**3**→**4**). The restoration of the two cysteine residues occurs by the intervention of thioredoxin reductase (TRR) and NADPH [8].

We recently discovered that the sulfur radical species, derived at different pHs from hydrogen sulfide (H_2_S), are able to transform 1,2-diols to alcohols via carbonyl reduction [13]. We hypothesized that the intermediate HSS^•2−^ is able to reduce the carbonyl moiety of ketones by a radical chain reaction. The HSS^•2−^ species is analogous to (CysSSCys)^●−^, which may be able to reduce ketones to corresponding alcohols under certain conditions. It has been shown that disulfide radical anions (RSSR)^●−^ are strong reductants, with the standard reduction potential (*E*^0^) of the RSSR/RSSR^●−^ redox couple in the range 1.40–1.60 V vs. NHE [14,15,16,17]. The reduction potential *E*^0^ (RSSR/RSSR^•−^) = −1.41 V found for the tripeptide glutathione disulfide is an indication of the reduction potential for the cystine residue in proteins [18]. Disulfide radical anions in proteins are stabilized by the tertiary protein structures and do not readily dissociate to thiyl radical and thiolate anion (cf. Structure **2** in Figure 1).

**Figure 2:**
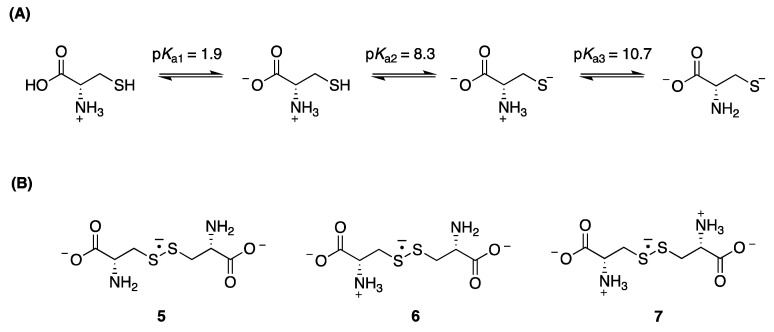
(**A**) The structure of cysteine, CysSH, and associated p*K*_a_ values; (**B**) Disulfide radical anion of cysteine (CysSSCys)^●−^ with 0 (**5**), 1 (**6**), and 2 (**7**) protonated amino groups.

Regarding cysteine (CysSH), which shows three p*K*_a_ values with the second one related to side chain thiol group (Figure 2A) [19], the equilibrium between (CysSSCys)^•−^and thiolate and thiol, namely:

CysS^●^ + CysS^−^ ⇆ (CysSSCys)^●−^ (1)

is a complex reaction studied in some detail by different experimental methods [18,20,21]. The forward rate constant (*k*_1_) was measured in the range pH 10–11 and found to be *k*_1_ = 1.2 × 10^9^ M^−1^s^−1^, whereas the reverse rate constant (*k*_−1_) strongly depends on whether the amino group is protonated or not, affecting the disulfide radical anion equilibrium (Equation (1)) and the reduction potential of CysSSCys/CysSSCys^●−^ redox couple (Figure 2B). The stability of (CysSSCys)^●−^ increases when protonated amino groups are present and is reflected in equilibrium constants *K*_1_: 438 M^−1^ (with zero protonated amino groups) (**5**), 317 M^−1^ (with one protonated amino group) (**6**), and 8900 M^−1^ (with two protonated amino groups) (**7**). Increases in stability of these species are indicated by progressively less negative reduction potentials: −1.50 V, −1.38 V, and −1.30 V, for 0, 1, and 2 protonated amino groups, respectively [18].

Here, we report the photochemical conditions found to transform ketones to corresponding alcohols by CysSH via a radical chain reaction involving (CysSSCys)^●−^ as reducing species. We also discovered that the reduction of two selected substrates proceeds via a dual radical chain mechanism. Furthermore, we applied time-resolved spectroscopy (pulse radiolysis) to generate (CysSSCys)^●−^ by reaction of CysS^−^ with HO^•^ radicals and provided the rate constants of both (CysSSCys)^●−^ and HO^•^ species with three cyclic ketones.

## 2. Results and Discussion

### 2.1. Ketone Reduction by the Photolysis of Cysteine-Containing Aqueous Solutions

#### 2.1.1. Cyclohexanone Reduction and Optimization Studies at Different pH Values

The photolysis (low-pressure Hg lamp, 5.5 W) of N_2_-saturated aqueous solutions of cyclohexanone (**8**, 8.3 mM) containing CysSH (18.3 mM) was monitored for up to 60 min and at various pH values (7.0, 7.5, 8.5, 9.6, 10.6, 11.5, 12.0, and 13.0) adjusted with 5% NaOH. In all experiments, cyclohexanol (**9**) was the only product. Table 1 reports the conversion of **8** to **9** at 30 and 60 min at each pH value. As shown in Table 1, cyclohexanol formation is achieved successfully at all pH values studied, reaching a maximum product yield of 96% at pH 10.6.

It is worth mentioning that other thiol derivatives such as 2-mercaptoethanol or *N*-acetylcysteine afford similar reductions but in lower yields (e.g., 51% and 56% after 60 min at pH 12, respectively) under identical experimental conditions.

#### 2.1.2. Reaction Mechanism: Initiation Steps for a Radical Chain Process

**Figure 3:**
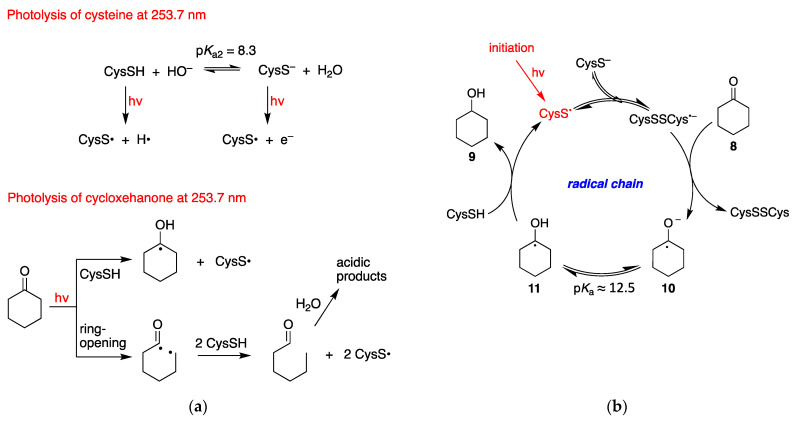
Proposed reaction mechanism for the reduction of cyclohexanone to cyclohexanol: (**a**) Possible initiation steps; (**b**) The radical chain reaction.

Based on the concentration of cyclohexanone and cysteine as well as their molar absorption coefficients, the light of 253.7 nm is absorbed by approximately 50% by each substrate. Light absorbed by the thiol/thiolate affords the thiyl radical together with H^•^/e^−^ (Figure 3a) [22]. Light absorbed by the cyclohexanone affords the S_1_ (n,π*) or T_1_ (n,π*) excited states. The T_1_ state can react with thiol [23], whereas both S_1_ and T_1_ states are reported to lead to a classical type I cleavage (ring-opening) [24,25], and we suggest that this biradical intermediate can react with thiol (Figure 3a).

At pH 10.6, where the yield of ketone reduction is higher (cf. Table 1), the CysS^−^ form is above 99%, being the p*K*_a_ value of SH moiety 8.3, although the concentration of CysSH is still ~150 μM. It is known that CysS^•^ adds reversibly to the parent anion to form the dimeric radical anion species, the forward reaction being close to the diffusion control rate (cf. Introduction). Therefore, due to the relatively high concentration of cysteine (18.3 mM), at higher pHs the main reactive species is (CysSSCys)^•−^. We propose that **8** is reduced to ketyl radical anion **10** by the RSSR^•−^, via single-electron transfer (SET). Subsequent protonation **10**→**11** from the aqueous medium and H-atom abstraction from CysSH affords the product **9**, completing the radical chain mechanism (Figure 3b). The low concentration of CysSH (~150 μM) is compensated by fast H-atom abstraction (*k*_H_ >10^8^ M^−1^ s^−1^ for analogous reactions [26,27]). It is worth mentioning that the p*K*_a_ values associated with the hydroxyl protons of ketyl radical **11** and alcohol **9** are ~12.5 and 16, respectively [28,29]. The overall process is perhaps facilitated by a concerted proton-coupled electron transfer (PCET) affording the ketyl radical **11** from **8** [30].

Next, we considered the reduction of **8** at pH 10.6 by changing the degassing conditions., i.e., N_2_O- instead of N_2_-saturated solutions. It is well-known that N_2_O-saturated solutions (~0.02 M of N_2_O) and e^−^_aq_ are efficiently transformed into HO^•^ radicals via Reaction 2 (*k* = 9.1 × 10^9^ M^−1^s^−1^) [31].

e^−^_aq_ + N_2_O + H_2_O → HO^•^ + N_2_ + HO^−^ (2)

In N_2_O-saturated conditions, the yields of cyclohexanol are 22% and 26% after 30 and 60 min of irradiation, respectively, in comparison to 86% and 96% of cyclohexanol after 30 and 60 min under N_2_-saturated conditions. This suggests that the presence of N_2_O strongly inhibits the radical chain reaction. Since (CysSSCys)^•−^ does not react with N_2_O, we suggest that the e^−^ generated by photolysis of CysS^−^ (cf. Figure 3a) undergo very fast hydration into hydrated electrons (e^−^_aq_) and then are trapped by N_2_O. This finding indicates that the addition of e^−^_aq_ to the carbonyl moiety of ketones is also an initiation step in the radical chain reaction described above.

Another important experimental observation is the decrease in pH during the reaction time (compare Columns 1 and 5 in Table 1). Tentatively, we suggest that after the ring-opening of the ketone triplet state and the reaction of biradical with thiol, the resulting aldehyde is the precursor of acidic products such as hydrated aldehyde. We could not obtain analytical evidences of by-products, probably due to their presence being below the detection limits.

#### 2.1.3. Other Substrates for Ketone Reduction at pH 10.6

**Figure 4:**
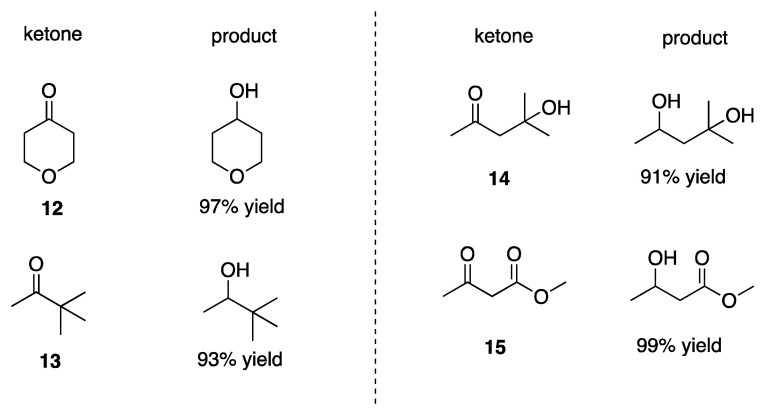
Reduction of ketones **12**–**15** to the corresponding alcohols. N_2_-saturated aqueous solutions of carbonyl compound (8.3 mM) cysteine (18.3 mM), pH adjusted to 10.6 at 42 ± 1 °C, were irradiated for 60 min. Yields by GC analysis based on products formation.

Considering the efficiency of the cyclohexanone reduction, the reduction of a few other ketones to the corresponding alcohols mediated by cysteine was further explored. Thus, under the optimized reaction conditions reported in Table 1 and at pH 10.6, ketones **12**–**15** all produced exclusively the corresponding alcohols, with yield ≥90% (Figure 4).

We demonstrated for the first time that the generation of ketyl radical anion via disulfide radical anion can be achieved. It is worth mentioning that: (i) SET reduction of ketones is a classical approach for the formation of ketyl radical anions, and such an approach has been used extensively in organic synthesis, as documented by the recently published tutorial review [32]; and (ii) ketyl radical anions formation have been typically carried out by means of dissolving metals such as Li, Na, and K, usually in liquid ammonia solution and in the presence of a proton source [33]. It can be envisaged that our bioinspired work will foster applications for SET methodology in aqueous environment.

#### 2.1.4. The Reduction of 2-Hydroxycyclohexanone and 2-Cyclopenten-1-one at pH 10.6 

For a better understanding of the reaction mechanism, we also considered the carbonyl reduction in 2-hydroxycyclohexanone (**16**) and 2-cyclopenten-1-one (**19**), having α-HO and internal α,β-unsaturated moiety, respectively.

**Figure 5:**
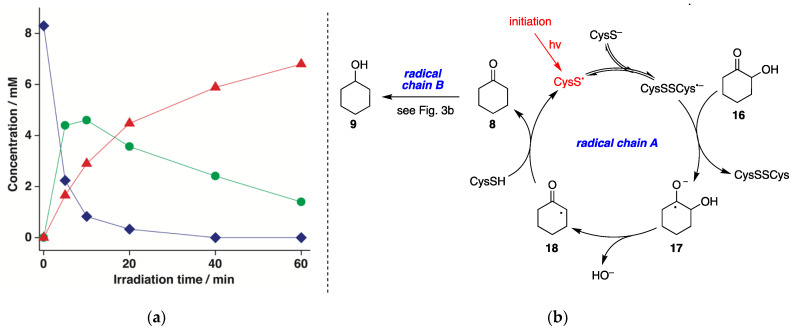
(**a**) Concentration of **16** (♦), **8** (●), and **9** (▲) vs. irradiation time for the photolysis of N_2_-saturated 2-hydroxycyclohexanone (**16**) aqueous solutions (8.3 mM) containing CysSH (18.3 mM), pH adjusted to 10.6, at 42 ± 1 °C; (**b**) Proposed reaction mechanism involving dual radical chain reactions A and B.

Photolysis (low-pressure Hg lamp, 5.5 W) of N_2_-saturated aqueous solutions of 2-hydroxycyclohexanone **16** (8.3 mM) containing CysSH (18.3 mM) was carried out for different times (up to 60 min) at pH 10.6. Cyclohexanone (**8**) and cyclohexanol (**9**) were formed as the only products. Figure 5a displays a graph with the disappearance of **16** (♦) and the formation of **8** (●) and **9** (▲) as a function of the reaction time, showing clearly that the reaction **16**→**9** occurs stepwise. The loss of **16** quantitatively matched the formation of **8** and **9**, with 17% and 82% yields, respectively, after 60 min. The mechanism is depicted in Figure 5b based on a dual radical chain process, starting from the reaction of (CysSSCys)^•−^ with **16** to produce the ketyl radical anion **17**. This species undergoes α,β-C−O scission and HO^−^ elimination, with the shift of the radical center to produce **18** [27], which completes the catalytic cycle by reacting with CysSH regenerating CysS^•^. The so-formed cyclohexanone (**8**) undergoes a second radical chain reaction, as described in Figure 3b.

**Figure 6:**
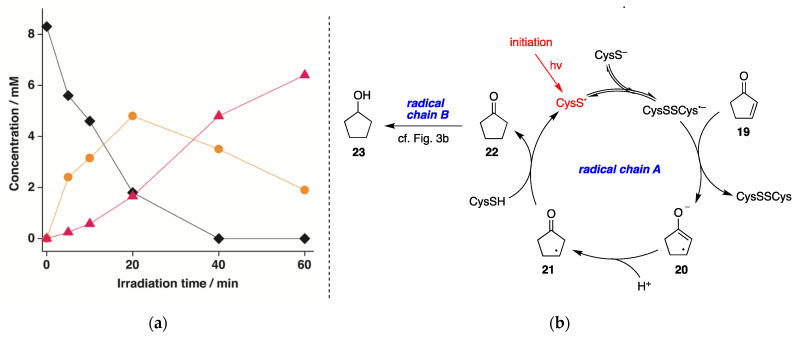
(**a**) Concentration of **19** (♦), **22** (●), and **23** (▲) vs. irradiation time for the photolysis of N_2_-saturated 2-cyclopenten-1-one (**19**) aqueous solutions (8.3 mM) containing CysSH (18.3 mM), pH adjusted to 10.6, at 42 ± 1 °C; (**b**) Proposed reaction mechanism involsving dual radical chain reactions A and B.

The reduction of 2-cyclopenten-1-one (**19**) was also explored under identical experimental conditions. The time profile of the reduction of (**19**) at pH 10.6 is reported in Figure 6a. In all experiments, cyclopentanone (**22**) and cyclopentanol (**23**) were formed as the only products, and again, the disappearance of **19** (♦) and the formation of **22** (●) and **23** (▲) as a function of the reaction time showed clearly that the reaction 1**9**→**23** occurs stepwise. The loss of **19** quantitatively matched with the formation of **22** and **23**, with 23% and 77% yields, respectively, after 60 min. The mechanism depicted in Figure 6b is based again on a dual radical chain process, starting from the single-electron transfer from (CysSSCys)^•−^ to **19** give the allyl-type radical **20**. This species undergoes protonation from the aqueous medium with double-bond shift to produce **21**, which completes the first catalytic cycle by reacting with CysSH regenerating CysS^•^. The so-formed cyclopentanone (**22**) undergoes a second radical chain reaction analogous to the one reported for cyclohexanone in Figure 3b.

### 2.2. Pulse Radiolysis Studies and Rate Constants of SET

Pulse irradiation of water leads to the primary reactive species e^−^_aq_, HO^•^, and H^•^, as shown in Reaction 3. The values in brackets represent the radiation chemical yield (*G*) in µmol J^−1^. In N_2_O-saturated solution (~0.02 M of N_2_O), e^−^_aq_ are efficiently transformed into HO^•^ radicals via Reaction 1 (*k* = 9.1 × 10^9^ M^−1^s^−1^), affording *G*(HO^•^) = 0.56 µmol J^−1^ [34].
H_2_O 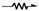 e^−^_aq_ (0.28), HO^•^ (0.28), H^•^ (0.06)(3)

The reaction of HO^•^ radicals with cysteine (100 mM) in the absence or in the presence of a ketone (up to 30 mM) was investigated in N_2_O-saturated solutions at pH 10.6. These experimental conditions were chosen in order (i) to maximize the formation of thiyl radicals (Reaction 4, *k*_4_ = (5.35 ± 0.82) × 10^9^ M^−1^s^−1^) [35], (ii) to minimize the quenching of HO^●^ by ketones (Reaction 5, unknown rate constants), (iii) to convert the thiol moiety of cysteine into thiolate ions, (iv) to maximize conversion of thiyl radicals into (CysSSCys)^●−^ (Reaction 1), and (v) to facilitate the electron transfer between (CysSSCys)^●−^ and ketones (Reaction 6). Furthermore, at pH 10.6, two forms of disulfide radical anion **5** and **6** are present at similar concentrations (Figure 2B).

HO^●^ + CysS^−^ → HO^−^ + CysS^●^ (4)

HO^●^ + R^1^R^2^C=O → H-atom abstraction (5)

(CysSSCys)^●−^ + R^1^R^2^C=O → CysSSCys + (R^1^R^2^C=O)^●−^ (6)

**Figure 7:**
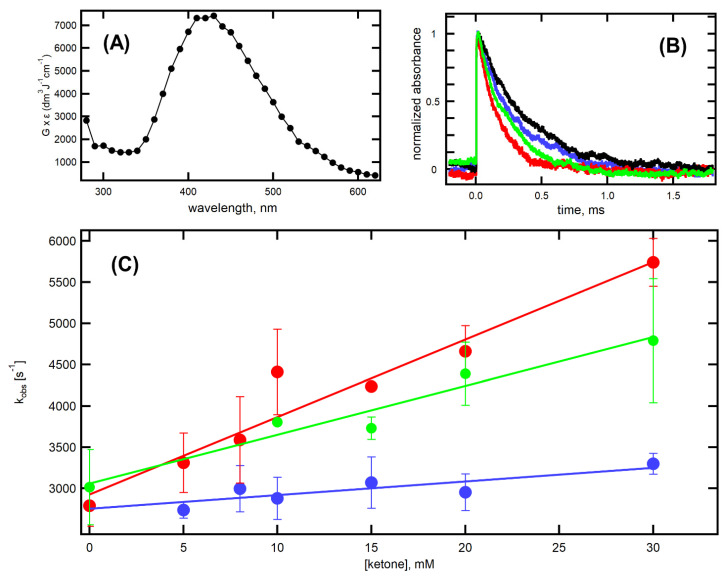
(**A**) Absorption spectrum of (CysSSCys)^●−^ recorded 10 μs after the electron pulse in N_2_O-saturated aqueous solutions at pH = 10.6 containing 100 mM of cysteine**.** (**B**) Normalized time profiles representing decay of transient absorption at λ = 420 nm, recorded after the electron pulse in N_2_O-saturated aqueous solutions at pH = 10.6, containing 100 mM of cysteine in the absence of ketones (black) and in the presence of 30 mM of **8** (blue), **16** (green) and **19** (red)**.** (**C**) Plots of the observed pseudo-first-order rate constants of the decay of the 420 nm absorption as a function of **8** (●), **16** (●) and **19** (●) concentration in N_2_O-saturated aqueous solutions at pH = 10.6, containing 100 mM of cysteine.

Transient absorption spectrum of (CysSSCys)^●−^ in the range 280–620 nm recorded 10 µs after the electron pulses is shown in Figure 7A, with the absorption maximum at 420 nm and in accord with previous reported ones [35]. The black traces in Figure 7B represent the decay of transient absorption at λ_max_ = 420 nm recorded after the electron pulse. The rate of decay of (CysSSCys)^●−^ was investigated by varying the concentrations of a ketone from 0 to 30 mM. We selected three ketones: cyclohexanone (**8**), 2-hydroxycyclohexanone (**16**), and cyclopentenone (**19**) for the time-resolved studies. The decay kinetics at various concentrations of **8**, **16**, and **19** were recorded at λ_max_, as shown in Figure 7B, for the presence of 30 mM of **8** (blue), **16** (green) and **19** (red). The pseudo-first-order rate constants of the decay of 420 nm absorption band were plotted as a function of ketone concentration (Figure 7C).

It is clearly seen that the pseudo-first-order rate constants measured at λ = 420 nm show a linear dependence on the concentration of **8**, **16**, and **19** in the full range of concentration studied (Figure 7C). The slopes in Figure 7C represent the second-order rate constants for the decay of (CysSSCys)^●−^ resulting from the reaction of (CysSSCys)^●−^ with **8**, **16**, and **19** (Reaction 6). The obtained values of *k*_6_ for three ketones are collected in Table 2. Both **16** and **19**, having α-HO and internal α,β-unsaturated moieties, increased the rate constant by 3.7- and 5.9-folds, respectively, due to extra stabilization of ketone radical anion.

By applying the common competition kinetics method [36], the rate constants for the reaction of HO^•^ radicals with ketones (Reaction 5) can also be estimated. To our best knowledge, these rate constants were not measured earlier. Indeed, from the plot of the reciprocal of [CysSSCys^●−^] versus the [CysSH]/[Ketone] ratio and considering *k*_4_ = (5.35 ± 0.82) × 10^9^ M^−1^s^−1^, we obtained the values of *k*_5_ reported in Table 2.

## 3. Materials and Methods

### 3.1. Materials and Instrumentation

All commercial chemicals were used as received unless otherwise noted. l-cysteine ≥ 98.5%, cyclohexanone ≥ 99.5%, cyclohexanol 99%, tetrahydro-4H-pyran-4-one 99%, tetrahydro-4-pyranol 98%, 3,3-dimethyl-2-butanone 97%, 3,3-dimethyl-2-butanol 98%, 4-hydroxy-4-methyl-2-pentanone 99%, hexylene glycol 99%, methyl acetoacetate 99%, methyl 3-hydroxybutyrate ≥ 95%, 2-hydroxycyclohexanone 99%, 2-cyclopenten-1-one 98%, cyclopentanone 99%, cyclopentanol 99%, 2-mercaptoethanol ≥ 99%, *N*-acetyl-l-cysteine European Pharmacopoeia Reference Standard, and phosphoric acid 85 wt% in water were obtained from Sigma-Aldrich (San Louis, MO, USA). Diethyl ether (HPLC grade) was obtained from Carlo Erba (Milan, Italy) and distilled before use. Water was purified with a Millipore system. Absorption spectra were recorded with a PerkinElmer Lambda 950 spectrophotometer (PerkinElmer, Shelton, USA). UV irradiations were performed in a micro-photochemical reaction assembly with quartz well (Ace Glass) using a 5.5 W cold cathode, low-pressure, mercury arc, and gaseous discharge lamp (corresponds to λ = 250–260 nm) made of double-bore quartz (Ace Glass). Ethyl ether extracts of the irradiated samples were analyzed by GC-MS (Thermo Scientific Trace 1300, Waltham, MA, USA) equipped with a 15m × 0.25mm × 0.25μm TG-SQC 5% phenyl methyl polysiloxane column, with helium as carrier gas, coupled to a mass selective detector (Thermo Scientific ISQ, Waltham, MA, USA).

### 3.2. Ketone Reduction

General procedure for the irradiation experiments. l-cysteine (13.3 mg, 0.11 mmol) was dissolved in 5 mL of N_2_-saturated water while continuously bubbling N_2_ in the photolysis apparatus. Cyclohexanone (4.9 mg, 0.05 mmol), tetrahydro-4*H*-pyran-4-one (5.0 mg, 0.05 mmol), 3,3-dimethyl-2-butanone (5.0 mg, 0.05 mmol), 4-hydroxy-4-methyl-2-pentanone (5.8 mg, 0.05 mmol), methyl acetoacetate (5.8 mg, 0.05 mmol), 2-hydroxycyclohexanone (5.7 mg, 0.05 mmol), or 2-cyclopenten-1-one (4.1 mg, 0.05 mmol) were added to the photolysis apparatus. The pH was adjusted to the specific value using a NaOH 5% *m/v* or a H_3_PO_4_ 5% *v/v* solution in deoxygenated water, and the final volume of the reaction was adjusted with N_2_-saturated water in order to reach 6 mL. The UV irradiation proceeded by inserting a 5.5 W low-pressure mercury arc lamp into a micro photochemical reaction assembly with quartz well, and N_2_ was smoothly flushing throughout the irradiation time at 42 ± 1 °C. After the irradiation was completed, 1 mL of irradiated sample was diluted with 200 mL of a NaCl-saturated aqueous solution and extracted with 6 × 0.5 mL diethyl ether. The organic layers (3 mL) were gathered, dried over Na_2_SO_4_ anhydrous, and analyzed by GC-MS.

GC-MS analyses of the diethyl ether extract of the irradiated samples were performed. The diethyl ether extracts were analysed by GC-MS equipped with a 15m × 0.25 mm × 0.25 μm TG-SQC 5% phenyl methyl polysiloxane column, with helium as carrier gas, coupled to a mass-selective detector following a specific oven program (see below). Injection volume was 0.5 μL. Identification of the reaction products was performed by comparison with the commercially available compounds by GC-MS analysis and spike experiments. Quantification of compounds was performed by multiple-point external standard calibration curves for each analyte of interest. For the reaction of cyclohexanone, 3,3-dimethyl-2-butanone, 2-hydroxycyclohexanone, and 2-cyclopenten-1-one, oven program was as follows: temperature started at 30 °C, maintained for 10 min, increased at a rate of 25.0 °C/min up to 250 °C, and held for 8 min. For the reaction of tetrahydro-4*H*-pyran-4-one, 4-hydroxy-4-methyl-2-pentanone, and methyl acetoacetate, oven program was as follows: temperature started at 40 °C, maintained for 1 min, increased at a rate of 5.0 °C/min up to 110 °C, increased at a rate of 20.0 °C/min up to 250 °C, and held for 8 min.

### 3.3. Pulse Radiolysis

The pulse radiolysis experiments were performed with the LAE-10 linear accelerator at the Institute of Nuclear Chemistry and Technology in Warsaw, Poland, with a typical electron pulse length of 10 ns and 10 MeV of energy. A detailed description of the experimental setup has been given elsewhere along with basic details on the equipment and its data collection system [37,38,39].

Absorbed dose per pulse was in the order of 3.5 Gy (1 Gy = 1 J kg^−1^). Experiments were performed with a continuous flow of sample solutions using a standard quartz cell with optical length 1 cm at room temperature (~22 °C). Solutions were purged for at least 20 min per 250 mL sample with N_2_O before pulse irradiation.

The dosimetry was based on N_2_O-saturated solutions of 10^−2^ M KSCN, which, following radiolysis, produces (SCN)_2_^●−^ radicals that have a molar absorption coefficient of 7580 M^−1^cm^−1^ at λ = 472 nm and are produced with a yield of G = 0.635 μmol J^−1^ [31].

## 4. Conclusions

The reduction of ketones via disulfide radical anion has been obtained for the first time in an aqueous environment, following a bioinspired process connected to the known mechanism of transformation of ribonucleotides to deoxyribonucleotides. Disulfide radical anion from cysteine, (CysSSCys)^●−^, is a very good 1e^−^ reductant, and experimental conditions were first set to obtain high-yield conversion of representative substrates. Our results can be considered as an example of bimolecular/associative reductant upconversion that has been recently reviewed [40]. Using time-resolved studies, we measured the rate constants of three cyclic aliphatic ketones to be in the range of 10^4^–10^5^ M^−1^s^−1^ at ~22 °C. Expansion of this methodology to other substrates and applications of disulfide radical anion in photoredox catalysis can be envisaged.

## Figures and Tables

**Table 1:** Photolysis (low-pressure Hg lamp, 5.5 W) of N_2_-saturated aqueous solutions of cyclohexanone (**8**, 8.3 mM) containing cysteine (18.3 mM) with formation of cyclohexanol (**9**) at different irradiation times and pH. ^1^.

pH (Initial)	Time (min)	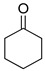 8 (mM)	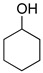 9 (mM)	pH (Final)
7.0	30	6.6	1.7	-
	60	5.1	3.2	6.0
7.5	30	5.5	2.8	-
	60	4.4	3.9	6.5
8.5	30	1.8	6.5	-
	60	1.0	7.3	7.5
9.6	30	1.9	6.4	-
	60	0.9	7.4	8.3
10.6	30	1.2	7.1	-
	60	0.3	8.0	9.5
11.5	30	1.7	6.6	-
	60	0.5	7.8	10.8
12.0	30	1.6	6.7	-
	60	0.6	7.7	11.5

^1^ Reaction temperature was constant at 42 ± 1 °C.

**Table 2:** Rate constants (M^−1^s^−1^) for the reactions of (CysSSCys)^●−^ and HO^•^ with the three cyclic ketones. (CysSSCys)^●−^ reacts by one-electron transfer and HO^•^ reacts by H-atom abstraction. ^1^.

*k* (M^−1^s^−1^)/Radical	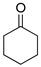 8	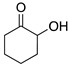 16	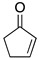 19
*k*_6_/(CysSSCys)^●−^	(1.6 ± 0.3) × 10^4^	(5.9 ± 0.5) × 10^4^	(9.4 ± 0.2) × 10^4^
*k*_5_/HO^•^	7.2 × 10^7^	8.6 × 10^7^	1.3 × 10^8^

^1^ At room temperature (~22 °C).

## Data Availability

The data presented in this study are available on request from the corresponding author.

## Sample Availability

Not available.
